# Ukraine conflict: Prioritizing lives and health

**DOI:** 10.1371/journal.pmed.1004007

**Published:** 2022-05-25

**Authors:** The PLOS Medicine Editors

**Affiliations:** Public Library of Science, San Francisco, California, United States of America

The Russian Federation’s “special military operation” in Ukraine began on February 24 and, at the time of writing, shows no sign of abating. As of April 28, the UN High Commissioner for Human Rights reported 6,009 civilian casualties of this brutal conflict (comprising 2,829 people killed and 3,180 injured), noting that the true figures are expected to be considerably higher [1]. Few will not have seen the shocking images broadcast from the country, and casualties among combatants on both sides can only be very substantial. This destructive and inhuman war can be unequivocally condemned and must cease as soon as possible.

Unfortunately, the events unfolding in Ukraine are far from unanticipated. Following the emergence of modern Ukraine from the dissolving Soviet Union in 1991, the country, with its minority Russian-speaking population, has maintained an uneasy coexistence with Russia. Emerging relationships with the European Union and its member states and the country’s aspirations to join NATO have caused obvious tensions. The resulting instability has led to several serious crises in recent years, and the current conflict can be seen as a dramatic escalation of existing hostilities following Russia’s annexation of the Crimea in 2014, as well as an indicator of Ukraine’s precarious situation in the region.

Even after just a few weeks of fighting, the tragic and unacceptable broader costs of the invasion by Russian forces, supported by neighboring Belarus, have been evident. Apparently widespread and indiscriminate use of artillery and missiles in populated areas has been enormously destructive and murderous, as illustrated by the bombardment of the city of Mariupol, close to the Russian border in the South of the country, including a reported air strike on a maternity hospital on March 9. In a joint statement, UNICEF, UNFPA, and WHO have noted that there have already been more than 100 attacks on healthcare entities since the start of the war in Ukraine, calling for an immediate cessation [2]. Deliberate violence against civilians, residential buildings, and health workers and facilities is indefensible in any setting.

Vast numbers of refugees have fled Ukraine during the conflict—estimated at more than 5.4 million people so far [3]—to safer countries. More than 2.9 million people have migrated to neighboring Poland, for instance, and 810,000 to Romania; some 650,000 people are believed to have entered Russia. Many more people will have been displaced internally in Ukraine. Within the country, health and medical provision is likely to have been severely disrupted by loss of medical facilities and personnel, by the additional demands of caring for the many casualties of the conflict, and by disruptions in medical supplies. There will be particular concern for vulnerable groups who may be unwilling or unable to seek safer spaces, including mothers and newborns, people with limited mobility or acute medical needs, and the elderly. Infectious disease outbreaks are an additional danger in such situations, and the Coronavirus Disease 2019 (COVID-19) is a particular concern given modest vaccination levels in the Ukrainian population [4]. The mental health impacts of the conflict will be pervasive and long lasting.

To add some context to the current situation, UNHCR estimates that 3 million people in Ukraine were in need of humanitarian support prior to the current war, with 850,000 people already displaced by conflict [5]. Needs for protection and humanitarian assistance will now be enormous and greatly increased both within and outside Ukraine. The Ukrainian government reports that 270,000 tons of humanitarian supplies have been provided since the beginning of the conflict, including food provision for some 12 million people [6]. Yet, with the duration, scale, and human toll of hostilities unknown, the magnitude of present and future needs can scarcely be guessed. Only a definitive end to the present conflict can bring about the conditions needed for people to feel safe and for the necessary medical provision to be made available to all people affected, and to Ukraine’s population as a whole.

Looking to the longer-term future, the conflict in Ukraine should not be allowed to distract attention from similarly disastrous wars elsewhere, including those in Syria and Yemen that have been going on for several years. The incalculable harms and costs of such conflicts mean that much greater efforts toward diplomacy and prevention are needed. Despite the sensible words from some leading figures in multinational fora, including the United Nations, the current balance of power can lead, at least overtly, to a paralysis in which the lives and well-being of vast populations are defenseless against the political, economic, and military agendas of stronger nations with unscrupulous and unaccountable leaders. Humankind deserves better from national and international leaders—perhaps lessons can be learnt from elements of cooperation, foresight, and altruism shown in some of the responses to COVID-19.
